# Analysis of the Antimicrobial, Cytotoxic, and Antioxidant Activities of *Cnidium officinale* Extracts

**DOI:** 10.3390/plants9080988

**Published:** 2020-08-04

**Authors:** Myung-Jin Lee, Min-Kyung Kang

**Affiliations:** 1 Department of Dental Hygiene, Division of Health Science, Baekseok University, Cheonan 330704, Korea; dh.mjlee@bu.ac.kr; 2 Department of Dental Hygiene, Hanseo University, 46 Hanseo 1-ro, Haemi-myun, Seosan-si 31963, Korea

**Keywords:** antibacterial agents, *Candida albicans*, *Cnidium*, *Streptococcus mutans*, flavonoids, polyphenols

## Abstract

This study analyzed the antimicrobial, cytotoxic, and antioxidant properties of *Cnidium* *officinale* (CO) extracts to confirm their antimicrobial activity toward oral microorganisms. The control group contained 0 μg/mL of CO, and the experimental groups contained 50, 100, 150, and 200 μg/mL of CO. To confirm the antibacterial activity of CO extracts against microorganisms in the oral cavity, an inhibition zone test, a colony-forming unit (CFU) analysis, an optical density (OD) evaluation, and a SEM (scanning electron microscopy) analysis were performed. A cytotoxicity test was also conducted to determine cell viability, and the contents of flavonoids and polyphenols were measured to analyze the extract components. In the control group, the growth inhibition zone increased, while the CFU and OD values decreased (*p* < 0.05). The SEM analysis confirmed that the number of microorganisms for both the microbes decreased. The cell viability was more than 80% in both the control and experimental groups, excluding the 200 μg/mL sample. The flavonoid and polyphenol contents in the experimental groups showed higher values than those of the control group. Therefore, the CO extract showed considerable antimicrobial activity toward both *Streptococcus mutans* and *Candida albicans*, suggesting that it may be used as a natural antimicrobial agent for dental applications.

## 1. Introduction

The oral cavity contains various kinds of bacteria and fungi [1,2]. Chemicals, such as antibiotics, are used to suppress oral bacteria [3,4]. However, antibiotics have harmful effects on the human body and can result in antibiotic resistance [5]. Because of their side effects, antibiotics are contraindicated for extended use to prevent oral infections or to suppress oral bacteria [6,7]. Furthermore, the strong chemicals that are added to mouthwashes are likely to remove resident oral bacteria along with the pathogens. Natural extracts are becoming more widely used in place of synthetic chemicals as part of efforts to specifically suppress the pathogens while retaining the resident oral bacteria [2,8]. These antibacterial agents are classified as organic or inorganic. The organic antibacterial agent can be further classified into a chemical synthetic product or a natural organic material [9]. Chemical synthetic products are inexpensive, have uniform composition, and can be mass-produced; however, the occurrence of toxicity, carcinogenesis, and mutagenesis have been reported in the human body [10,11]. The natural, organic variety have specific limitations arising from the dependence of their composition and ingredients on manufacturing conditions; however, they have the advantage of less residual toxicity and very few side effects [12]. In particular, since natural extracts have long been used as medicinal products, biotechnology has facilitated the evolution of a competitive industry through the development of functional products and other pharmaceutical and antibacterial products [13,14]. Recently, various studies on natural extracts that are used as antioxidants have been conducted to study their suppression of free radicals. *Cnidium officinale* (CO), an herbal medicine in Korea, is a perennial plant belonging to the family Umbelliferae. It is largely used for physiological treatments and as a traditional analgesic and anti-inflammatory medicine in Asia; it is known as an important medicine used to overcome various diseases. [15,16]. The rhizome of CO has various pharmacological effects, such as antibacterial, antifungal, angiogenic, and sedative properties [15,16,17]. In addition, studies on aerial parts as well as rhizomes of CO have also reported its ability to detoxify oxidizing substances and other toxic substances generated in the body, its inhibitory activity towards mutations leading to cancer, and its antifungal activity against phytopathogenic fungi [18,19,20,21]. Accordingly, CO is known to have antibacterial activity against various microorganisms, such as bacteria and fungi; however, there is a paucity of research on its effects on oral microorganisms. *Streptococcus mutans* (*S. mutans*) and *Candida albicans* (*C. albicans*) have especially been investigated in this study because they are known as representative microorganisms involved in various oral diseases. Therefore, the purpose of this study is to investigate the antimicrobial, cytotoxic, and antioxidant activities of CO extracts in order to determine their properties and potential effects on oral microorganisms.

## 2. Results

### 2.1. Inhibition Zone Test

**Figure 1:**
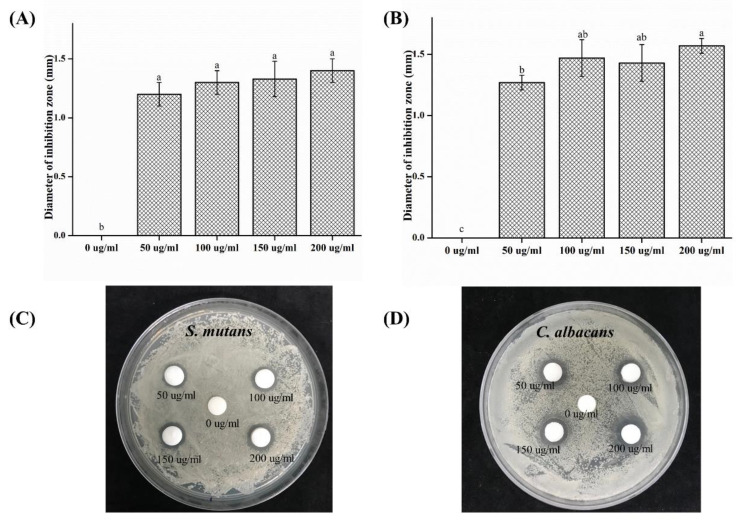
Comparison of the antimicrobial properties between different groups. The diameters of the zone of inhibition on the agar plate were measured for (**A**) *S. mutans* and (**B**) *C. albicans*. Images of the inhibition zone test on agar plates with (**C**) *S. mutans* and (**D**) *C. albicans*. Different letters above bars indicate significant differences (*p* < 0.05).

The antimicrobial activity of *S. mutans* and *C. albicans* toward different concentrations of CO was tested through the inhibition zone test, and the results are shown in Figure 1. The antimicrobial activity against *S. mutans* and *C. albicans* showed a significant difference in all experimental groups compared to the response of the 0 μg/mL (*p* < 0.05) solution of the control group.

### 2.2. Colony-Forming Unit (CFU) Counts

**Figure 2:**
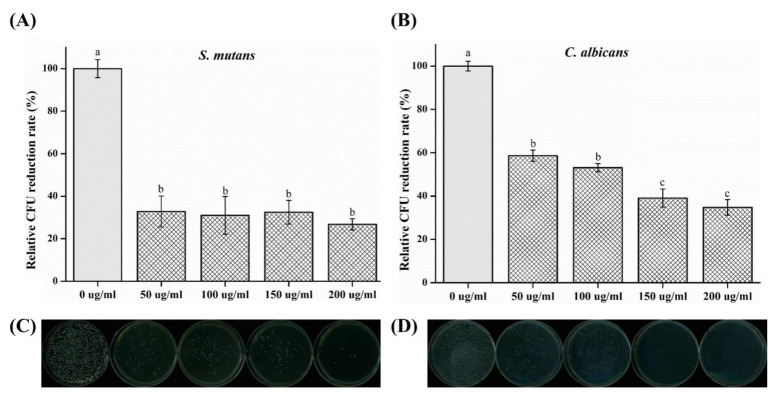
Relative colony-forming unit (CFU) reduction rate for (**A**) *S. mutans* and (**B**) *C. albicans*. (**C**,**D**) Images of agar plates with (**C**) *S. mutans* and (**D**) *C. albicans*. Different letters above bars indicate significant differences (*p* < 0.05).

The relative colony-forming unit (CFU) reduction rate is shown in Figure 2. In the case of *S. mutans*, the CFUs were significantly decreased in all experimental groups as compared to those of the control (*p* < 0.05) group. In the case of *C. albicans*, the extracts’ ability to inhibit the growth of this fungus increased with an increase in the concentration of CO, and a significant difference was observed between the groups with and without CO (*p* < 0.05).

### 2.3. Optical Density (OD)

**Figure 3:**
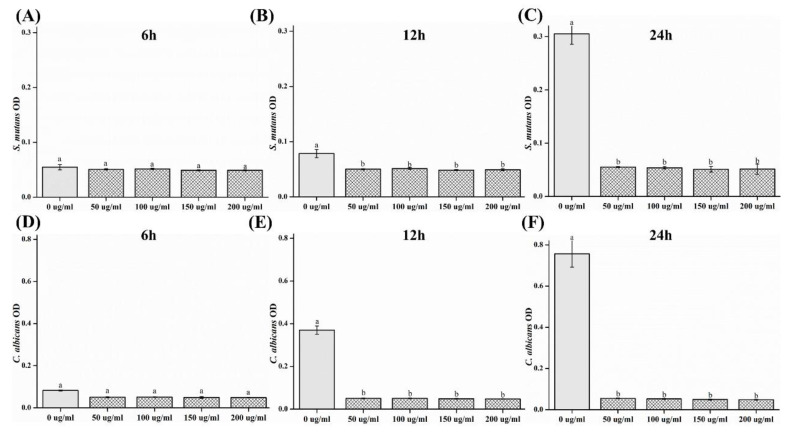
Comparison of optical density (OD) of (**A**) *S. mutans*, 6 h; (**B***) S. mutans*, 12 h; (**C**) *S. mutans*, 24 h; (**D**) *C. albicans*, 6 h; (**E**) *C. albicans*, 12 h; and (**F**) *C. albicans*, 24 h. Different letters above bars indicate significant differences (*p* < 0.05).

The optical density (OD) results are shown in Figure 3. The ODs of both *S. mutans* and *C. albicans* were observed to decrease significantly after 12 and 24 h in the samples containing CO extracts (*p* < 0.05).

### 2.4. Scanning Electron Microscopy (SEM)

**Figure 4:**
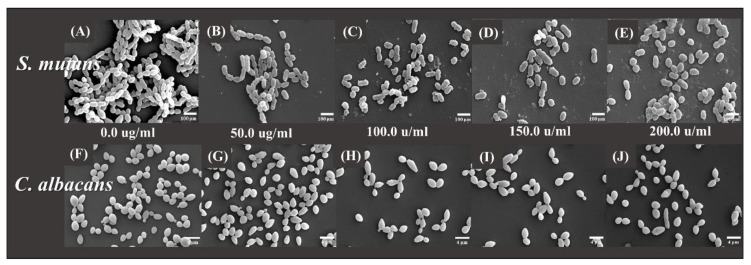
Field-emission scanning electron microscope images of *S. mutans* on (**A**) 0, (**B**) 50, (**C**) 100, (**D**) 150, and (**E**) 200 μg/mL of *Cnidium officinale* (CO) extracts (scale bar = 100 μm) and *C. albicans* on (**F**) 0, (**G**) 50, (**H**) 100, (**I**) 150, and (**J**) 200 μg/mL of CO extracts (scale bar = 4 μm).

The morphologies of the microorganisms are shown in Figure 4. It was confirmed that the number of microorganisms decreased in the CO experimental groups as compared to that of the control group. In addition, in *S. mutans*, it was observed that the chain of bacteria had been shortened and the shape had changed irregularly.

### 2.5. Biological Properties

**Figure 5:**
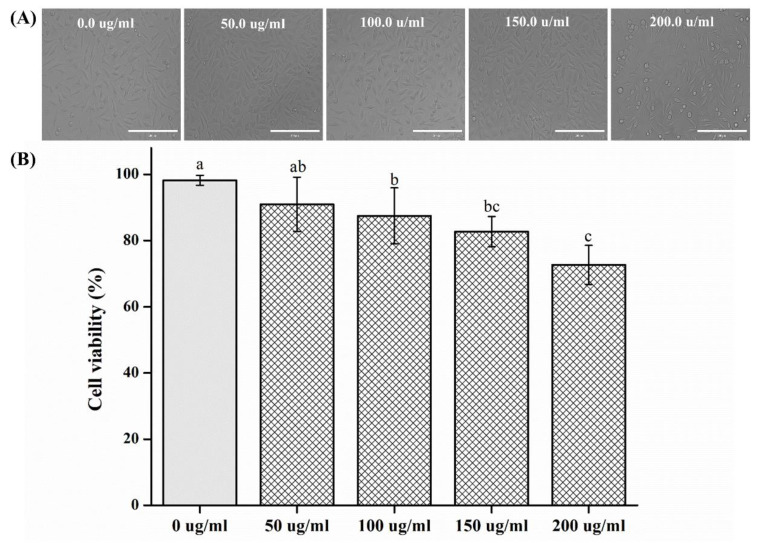
(**A**) Representative microscopic images of L929 cells: 0, 50, 100, 150, and 200 μg/mL at 20× magnification. (**B**) Comparison of cell viability among different groups. Different letters above bars indicate significant differences (*p* < 0.05).

The cytotoxicity test results are shown in Figure 5. Cell shape was normal in all the experimental groups. There was no significant difference in cell viability observed in the groups with concentrations between 0 and 50 μg/mL (*p* > 0.05). In the groups with concentrations above 100 μg/mL, there was a significant difference in comparison to the 0 μg/mL group (*p* < 0.05).

### 2.6. Measurement of Polyphenol and Flavonoid Contents

**Figure 6:**
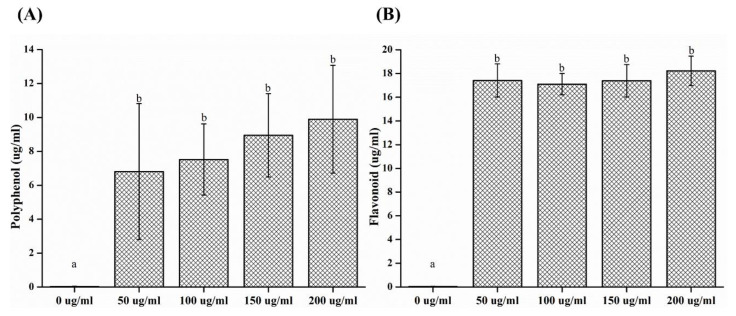
(**A**) Polyphenol and (**B**) flavonoid contents of CO extracts. Different letters above bars indicate significant differences (*p* < 0.05).

The phenolic compounds in plants have been found to be related to their antimicrobial activity. The concentrations of polyphenol and flavonoids in the CO extract were confirmed. The polyphenol contents of CO at 0, 50, 100, 150, and 200 μg/mL were 0.03 ± 0.01, 6.81 ± 4.01, 7.52 ± 2.10, 8.95 ± 2.45, and 9.90 ± 4.18 µg/mL, respectively. The flavonoid contents of CO at 0, 50, 100, 150, and 200 µg/mL were 0.04 ± 0.01, 17.40 ± 1.40, 17.10 ± 0.90, 17.39 ± 1.36, and 18.22 ± 1.25 µg/mL (Figure 6). There was a significant difference between the results of the control and experimental groups (*p* < 0.05).

## 3. Discussion

In this study, the antimicrobial, cytotoxic, and antioxidant activities of CO extracts were investigated. The oral cavity is colonized by microflora [22] consisting of bacteria, viruses, and fungi. Approximately 500 species of bacteria exist in the oral cavity [3]. In particular, a fungus known as *C. albicans* inhabits the oral cavity; if a large number of this type of fungus exists, an infection called candidiasis becomes highly probable [2]. *S. mutans* is reported to be the primary causative agent for the formation of dental caries owing to the synthesis of glucans from sucrose-secreting glucosyltransferases [23]. The inhibition of microbial activity can result in a reduction of reproduction and pathogenesis [1,4]. Before using natural extracts for oral applications, their antimicrobial effects on microorganisms must be verified [22]. *S. mutans* causes caries, and an organic acid is formed during glycolysis [1,23]. The acidic environment that it creates further promotes the attachment of *C. albicans* to the oral mucosa and dentures [2]. In this study, we attempted to confirm the antimicrobial activity of the oral microorganisms *C. albicans* and *S. mutans* [24]. To confirm the concentration-dependent antimicrobial activity of CO extracts, the inhibition zone, number of colonies, and OD values of *S. mutans* and *C. albicans* at 0, 50, 100, 150 and 200 mg/mL concentrations were studied. The results showed a significant difference with respect to that of the control group. Accordingly, the antibacterial activity of CO was verified. It was demonstrated that the CO extracts showed antibacterial activity against both microorganisms, *S. mutans* and *C. albicans*. Based on these results, CO is believed to be helpful in preventing or improving oral diseases. However, various clinical isolates of the same microorganism can differ in properties including an ability to form biofilm, and microbes attached to a surface can display altered susceptibility to antibiotics or antifungal agents. Since the standard reference microorganism was selected in this study, the experiment has the limitation of the lack of testing for the potential inhibitory properties of the extract against oral isolates.

The composition of the CO extract was also investigated in this study. A large number of research studies have been performed on the antibacterial substances that are present in natural products [12], and numerous studies have considered the antibacterial properties of plants. The majority of these studies were focused on the presence of phenolic compounds, which are secondary metabolites found in approximately 8000 species [25]. These polyphenolic compounds exhibit a natural antioxidant activity [25,26]. Therefore, many research studies are actively being conducted on plants in order to search for new substances with natural and functional effects [16]. The research has primarily focused on methods to separate and use substances that are safe for the human body and on the natural products that have a high antibacterial activity and are safe for consumption [25]. Phenolics, the representative phytochemical compound, have an aromatic ring with one or more hydroxyl substituents. The phenolic compounds that can be recognized as the soft material contained in plants have excellent anti-inflammatory, antitumor, atherosclerotic, anticancer, and antibacterial effects. The functional effects of CO as a natural product were verified in our study [25,27]. The antioxidant activity of the natural extracts can be attributed to various components, particularly the total phenolic and flavonoid content present in the extracts [19,27]. These results can be related to phenolic compounds such as polyphenols and flavonoids [27] that exhibit antibacterial activity [21]. The antioxidant effects of the natural extracts are mediated by various ingredients, mainly total phenols [25] and the total flavonoid content. Previous studies have also demonstrated the antioxidant activity and the effect of the phenolic components of CO extracts [25,27]. In this study, there was a limitation in investigating the content of polyphenols and flavonoids. Indeed, additional experiments with other components or metabolites related to the antioxidant effect should be considered in future studies. Cytotoxicity was tested to confirm the safety of the natural CO extracts. The MTT method, widely known as a cytotoxicity test, was used to evaluate the toxicity toward cells [2]. This is a quantitative method that has been mainly used as a primary assay for determining chemical cytotoxicity [2]. All experimental groups showed a high cell survival rate of above 60%. In these experiments, the CO extracts showed no cytotoxicity and established their potential as a material with antimicrobial properties; however, there were limitations, such as a short experimental time period. Therefore, additional evaluations and experiments, such as formulation studies, are required. For applications in dentistry, it is necessary to conduct follow-up studies that can classify component ratios and observe the effects over time. It is necessary to conduct further studies to clarify the mechanisms of antibacterial action and long-term effects for clinical applications [1,2]. For use in the oral cavity, in vivo experiments are necessary in the future, as antibacterial effects may not be accurately reproducible because the presence of saliva and the process of swallowing are hard to model [4,23].

## 4. Materials and Methods

### 4.1. Materials

CO leaves (Sancheong, Gyeongsangnam-do, Korea) were commercially purchased at an herbal shop that sells the identified *Cnidium officinale* that compared to the standard herbal specimen provided by the National Institute of Food and Drug Safety Evaluation. After crushing 500 g of CO leaves, they were placed in a solution of 70% methanol and extracted at room temperature for 24 h. The extracts were first filtered (using Filter paper #2, Whatman, UK). The filtered extract was concentrated using a vacuum evaporator (Vacuum Evaporator, ETELA, Japan). The concentrated extract was pulverized using a freeze dryer (Freeze Dryer, Ilshin Lab, Korea). The pulverized powder was added to dimethyl sulfoxide (DMSO; Amresco, Solon, OH, USA) to prepare solutions of 0 (control), 50, 100, 150, and 200 μg/mL concentrations of CO extract.

### 4.2. Microbial Preparation

For microbial analysis, the growth inhibitory effect of the extracts was analyzed. The strains of microbes used in this experiment were *Streptococcus mutans* (ATCC 25175) and *Candida albicans* (ATCC 10231). *S. mutans* was cultured in a brain heart infusion (BHI) broth (Becton Dickinson and Co., MD, USA) and *C. albicans* in a yeast and mold (YM) agar medium (Becton Dickinson and Co., Franklin Lakes, NJ, USA); both were incubated at 37 °C for 24 h.

### 4.3. Inhibition Zone Test

Herein, 100 μL of the microbial culture suspension (1 × 10^4^ cells/mL) was spread uniformly on the BHI and YM agar plates. A filter paper disk, with the same diameter as the disk-shaped sample, was placed on the surface of the agar plate, soaked with 20 μL of the sample. The plates were incubated for 24 h at 37 °C, and the inhibition zones around each sample were measured using a Vernier caliper (Mitutoyo, Kawasaki, Japan).

### 4.4. OD

The CO extract solutions were prepared with different concentrations of 50, 100, 150, and 200 μg/mL CO extract. The microbial culture fluid was diluted such that the OD 600 value was between 0.4 and 0.6. The sample solution and bacterial culture were mixed at a ratio of 9:1 and incubated at 37 °C for 24 and 48 h. The inhibitory effect of the extract solutions was measured based on the OD values of each using an ELISA plate reader (Epoch, BioTeck, Winooski, VT, USA) at 600 nm.

### 4.5. CFUs

The sample solution and microbial culture (1 × 10^5^ cells/mL) were mixed at a ratio of 1:1; 100 μL of this mixture was spread onto the BHI and YM agar plates and incubated at 37 °C for 24 h. Subsequently, the total number of colonies was counted.

### 4.6. SEM

Herein, 1 mL of microbial suspension (1 × 10^5^ cells/mL) was placed onto a 24-well plate and incubated at 37 °C for 24 h. For microscopic examination, the microbe was placed in 2% paraformaldehyde–glutaraldehyde in 0.1 M PBS for at least 30 min at room temperature. The samples were postfixed with 1% OsO_4_, which was dissolved in 0.1 M PBS for 2 h. Thereafter, they were dehydrated in an ascending gradual ethanol series, treated with isoamyl acetate, and subjected to a critical point drying process (LEICA EM CPD300; Leica, Wien, Austria). Subsequently, the samples were coated with Pt (5 nm) using an ion coater (ACE600; Leica) and examined and photographed using a scanning electron microscope (FE-SEM; Merin, Carl Zeiss, Oberkochen, Germany) at 2 kV.

### 4.7. Biological Properties

According to the MTT cytotoxicity test method of ISO 10993-5, the number of L929 cells per well was adjusted to 1 × 10^4^, and 100 μL was dispensed onto the wells and cultured for 24 h. After incubation, 100 μL of the natural extract diluted to various concentrations was applied to the cells for 24 h. As a control, RPMI 1640 without the natural extract was used. After application, the extract was discarded and washed with 100 μL of DPBS (Gibco BRL, Life Technologies, NY, USA). Subsequently, DPBS was removed and 50 µL was added per well and cultured with 1 mg/mL of MTT (Sigma, UK) for 2 h. In order to dissolve the as-formed MTT (3-(4–dimethylthiazol-2-yl) -2,5-diphenyltetrazolium bromide) formazan, 100 mL of isopropanol (Sigma, UK) was added to 100 µL/well and reacted for 20 min. Thereafter, the absorbance was measured at 570 nm on a spectrophotometer and analyzed. The result was normalized to 100% of the MTT reduction rate of the control group and expressed as a percentage. For the microscopic observation, the images of the L929 cells exposed to sample extracts were observed using an EVOS FL microscope (Advanced Microscopy Group USA Ltd, Mill Creek, WA, USA) at 20× magnification.

### 4.8. Measurement of Polyphenol and Flavonoid Contents

The polyphenol content was measured by immersing the specimen in distilled water using a solution eluted in a water bath at 37 °C for 7 days (similar to the MTT elution method). Then, 50 µL of the sample solution was added to 650 µL of distilled water. Subsequently, 50 µL of the Folin–Denis reagent was added and allowed to react at room temperature for 3 min, after which 100 µL of 10% Na_2_CO_3_ saturated solution was added, and 150 µL of distilled water was used to adjust the final volume to 1 mL. After mixing and incubating the reaction for 1 h in a water bath at 37 °C (dark room), the absorbance was measured at 725 nm using a UV/VIS spectrometer (X-ma 1200 Spectrophotometer, Human, Korea). A standard curve was constructed using gallic acid, and the polyphenol content was calculated by extrapolation. For the determination of the flavonoid content, 100 µL of the sample solution was added to 1 mL of diethylene glycol. Thereafter, 100 µL of 1N NaOH was added and allowed to react in a water bath at 37 °C for 1 h. The absorbance was measured at 725 nm using a UV/VIS spectrometer (X-ma 1200 Spectrophotometer, Human, Korea). The standard curve was constructed using naringin (Sigma Aldrich, St. Louis, MO, USA), and the flavonoid content was calculated by extrapolation.

### 4.9. Statistical Analyses

All statistical analyses were conducted using the SPSS 23 software program (IBM Corp, Armonk, NY, USA), and the level of significance was set at *p* < 0.05. The results among different groups were analyzed by one-way analysis of variance (ANOVA) followed by Tukey′s post hoc test.

## 5. Conclusions

In this study, antimicrobial, cytotoxic, and antioxidant activities of CO extracts were analyzed, and the following results were obtained:

1. In the inhibition zone test, the growth inhibition zone was observed to increase; the inhibition zones of both strains of microbes showed significant differences as compared to that of the control (*p* < 0.05) group.
2. CFU measurement showed a reduction in the number of colonies; both the strains of microbes demonstrated a significant difference in CFU reduction as compared to that of the control (*p* < 0.05) group.
3. The OD values decreased, such that both the strains of microbes showed a significant difference in OD as compared to that of the control group after 12 and 24 h (*p* < 0.05).
4. SEM analysis demonstrated a tendency for both the microbial numbers to decrease as compared to that of the control group.
5. In cell viability studies, no significant differences were observed in the control group and the 50 μg/mL solution (*p* > 0.05); significant differences in cell viability were observed in 100, 150, and 200 μg/mL groups as compared to that of the control (*p* < 0.05) group.
6. In the analysis of polyphenol and flavonoid contents, significant differences were observed between the control and experimental groups (*p* < 0.05).

The results of this study demonstrate that CO extracts have the potential to minimize antimicrobial infections. These results suggest that CO extracts may have clinical applications in dentistry.
